# How Machine Learning Can Help Close Evidence Gaps for Drug Safety in Pregnant Women

**DOI:** 10.2196/101042

**Published:** 2026-05-27

**Authors:** Michelle Falci

**Keywords:** drug safety in pregnancy, machine learning model, pregnancy clinical trial, machine learning drug safety, artificial intelligence drug safety

## Abstract

Women, especially pregnant women, have historically been excluded from clinical research. In this *News and Perspectives* article, JMIR Correspondent Michelle Falci reports on how advances in data analytics may help bridge evidence gaps.

**Key Takeaways:**

- Using large datasets and machine learning, researchers aim to close evidence gaps in drug safety created by excluding pregnant women from clinical research.
- An emphasis on interpretability and causal inference prevents relying on machine learning models that function as “black boxes,” drawing conclusions without showing their work.

In response to birth defects caused by medications like thalidomide, the US Food and Drug Administration (FDA) advised in September 1977 that “any premenopausal woman capable of becoming pregnant” be excluded from phase 1 and 2 clinical trials. Despite initiatives like the National Institutes of Health (NIH) Revitalization Act of 1993, which mandated the inclusion of women and other minorities in all NIH-funded research, the FDA’s 1977 guidance spurred a decades-long trend of women—especially pregnant women—being excluded from clinical research on a global scale.

## The Evidence Gap

According to an online survey of over 1200 women, this widespread exclusion leaves the 79% of women who take at least one medication during pregnancy to make decisions about their medication without sufficient evidence. Of that 79%, 63.2% made at least one medication change, and 36.5% of women who stopped their medication or lowered or missed a dose did so independent from their health care provider’s input.

Citing concerns about potential risks among pregnant women and “distrust of women to prevent pregnancy,” clinical trial developers admit that women are underrepresented in medical research.

Globally, 4% of clinical trials over the past decade allowed inclusion of pregnant women. To address this issue, the World Health Organization established a task force to achieve “timely and ethical inclusion of pregnant and breastfeeding women in clinical research for medical health products by 2030.”

Other efforts to determine whether a medication is safe for pregnant or breastfeeding women, like the FDA’s Pregnancy and Lactation Labeling Rule (PLLR), do not fully close the evidence gap. Of the 290 medications newly approved by the FDA between January 2010 and December 2019, all products submitted after June 20, 2015 were PLLR compliant, but about one-third of medications submitted between 2010 and 2015 were not in PLLR format by the June 30, 2019 deadline, and less than 20% of new product labels included data on human pregnancy and lactation.

**Figure:**
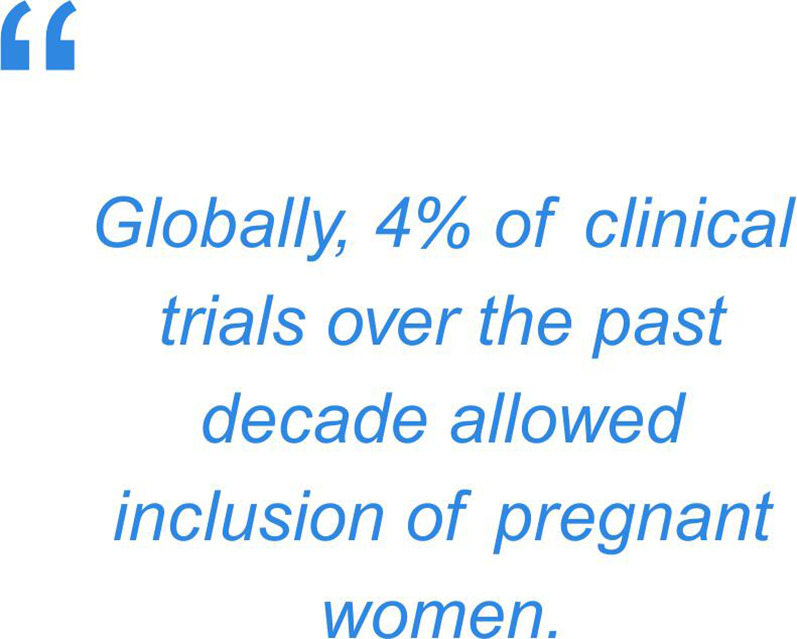


Researchers have tried to quantify the effects of including versus excluding pregnant women from randomized controlled trials (RCTs). In the case of thalidomide, had there been an RCT with 200 participants, about 33 children would have experienced adverse events during the trial, and this knowledge would have prevented approximately 8000 thalidomide-related birth defects, equivalent to 99.6% of all thalidomide-related birth defects from 1956 to 1962. This same study analyzed what would have happened if COVID-19 vaccine trials included pregnant participants and projected that 20% of all COVID-19–related maternal deaths and stillbirths between March and November 2021 could have been prevented if there were pregnant participants in the vaccine trials.

Decades of widespread exclusion have created a deep evidence gap for pregnant women and the clinicians who care for them, but there is one way that researchers are addressing the issue: analyzing large datasets with the help of machine learning to make recommendations on drug safety.

## How Data Mining and Machine Learning May Close Evidence Gaps

Almut G Winterstein, RPh, PhD, FISPE, is a principal investigator of the BOOST-HP project. The project includes three main stages: first, data mining to find links between medication exposure and outcomes, then triaging statistical signals to determine the plausibility of each pathway, and finally, evaluating which signals may have the highest clinical significance.

Winterstein’s tree-based approach to data mining is fully transparent—she and her team can see all of the decision pathways and variables that drive each signal, allowing them to trace how specific drug exposures are linked to outcomes.

“It is not one of these AI models where you are really in a black box and you don’t even know what the system is doing,” Winterstein says. “We can still follow very closely what is happening.”

The opacity of certain artificial intelligence (AI) models concerns Winterstein because she has seen them ignore core epidemiological principles, such as the requirement that exposure precedes outcome, she explains.

“I think we’re doing a disservice if we overuse this method without thinking about what is needed to basically establish causality, and then the methods will become discredited and nobody wants to deal with it anymore,” Winterstein says. “I think it is important for people who are very strongly on the AI side to collaborate with people who come from the causal inference epidemiology side and put those two together.”

Cristina Longo, PhD, leads the BIONIC study, which combines causal inference and machine learning to assess the safety and efficacy of biologic medications in pregnant women with chronic inflammatory conditions and then determine which women and children are at highest risk of poor outcomes.

“Our studies are very much based on frameworks that allow us to estimate causal effects, which is important for this type of work because AI helps identify risk-stratified patients. It can’t tell us what the causes are,” Longo says.

**Figure:**
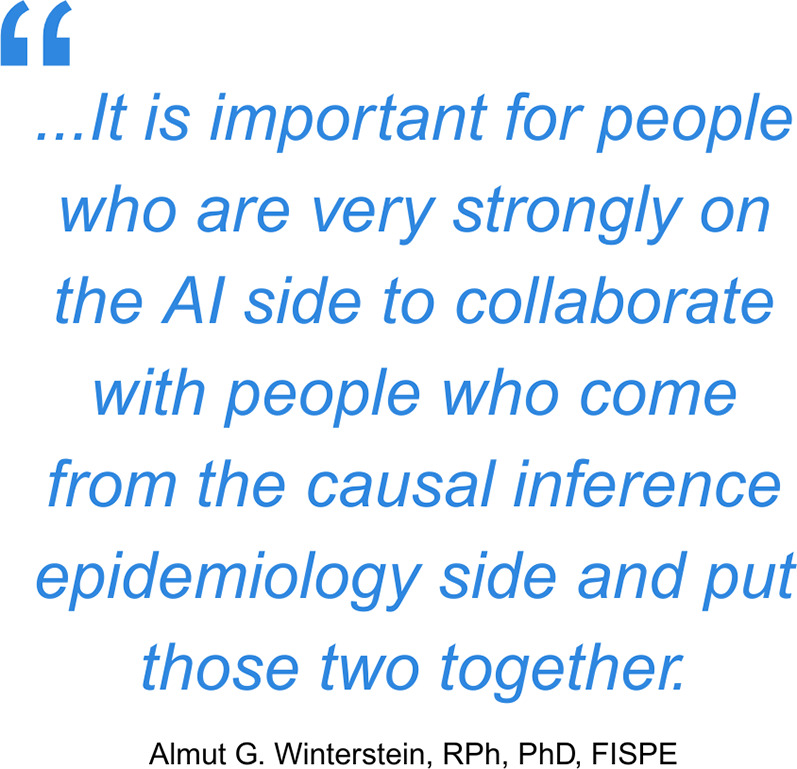


Leveraging large datasets applies to other facets of maternal health care beyond medication safety. In one study, researchers proposed that “digital twin” models of pregnant patients that are updated with real-time imaging and laboratory data can help predict outcomes for common complications during pregnancy.

Access to large and detailed datasets, Longo argues, is necessary to properly train machine learning models.

“Canada has a universal health care system. We have access to what people do at their physician visits and what types of medications they’re taking. But eventually what we’re hoping to achieve is access to more data: demographics, gender-based information, and socioeconomic status. These are all very important for AI models because we need to be able to make sure that they’re fair,” she says.

## The Future

The need for more data is not the only limitation of using AI to close the evidence gap created by the widespread exclusion of pregnant women from RCTs. The “black box” effect Winterstein mentioned is a risk for any project that relies on a model that does not allow researchers to clearly trace how inputs are translated into predictions. When working with a black box model, researchers cannot easily evaluate whether predictions are based on causal links or whether they are biased.

Some machine learning models for drug safety in pregnancy provide partial interpretability by including enhanced explainability mechanisms or apply post hoc interpretability methods even when inherently relying on a black box model.

“A lot of people are using AI in a way that it helps them, but they still want to keep control. And I think the same applies to research. At the end, in science, you still need to keep control,” Winterstein says.

Despite challenges with interpretability, researchers are optimistic that machine learning models can improve risk assessment for medication safety in pregnant women. By identifying safety signals and high-risk subgroups, these models may enable more targeted and ethically designed trials that include pregnant participants earlier in the evidence-generation process.

